# The Chromosomal Proteins JIL-1 and Z4/Putzig Regulate the Telomeric Chromatin in *Drosophila melanogaster*

**DOI:** 10.1371/journal.pgen.1003153

**Published:** 2012-12-13

**Authors:** Rute Silva-Sousa, Elisenda López-Panadès, David Piñeyro, Elena Casacuberta

**Affiliations:** Institute of Evolutionary Biology, Institut de Biologia Evolutiva (CSIC-UPF), Barcelona, Spain; Fred Hutchinson Cancer Research Center, United States of America

## Abstract

*Drosophila* telomere maintenance depends on the transposition of the specialized retrotransposons *HeT-A*, *TART*, and *TAHRE*. Controlling the activation and silencing of these elements is crucial for a precise telomere function without compromising genomic integrity. Here we describe two chromosomal proteins, JIL-1 and Z4 (also known as Putzig), which are necessary for establishing a fine-tuned regulation of the transcription of the major component of *Drosophila* telomeres, the *HeT-A* retrotransposon, thus guaranteeing genome stability. We found that mutant alleles of *JIL-1* have decreased *HeT-A* transcription, putting forward this kinase as the first positive regulator of telomere transcription in *Drosophila* described to date. We describe how the decrease in *HeT-A* transcription in *JIL-1* alleles correlates with an increase in silencing chromatin marks such as H3K9me3 and HP1a at the *HeT-A* promoter. Moreover, we have detected that *Z4* mutant alleles show moderate telomere instability, suggesting an important role of the JIL-1-Z4 complex in establishing and maintaining an appropriate chromatin environment at *Drosophila* telomeres. Interestingly, we have detected a biochemical interaction between Z4 and the HeT-A Gag protein, which could explain how the Z4-JIL-1 complex is targeted to the telomeres. Accordingly, we demonstrate that a phenotype of telomere instability similar to that observed for *Z4* mutant alleles is found when the gene that encodes the HeT-A Gag protein is knocked down. We propose a model to explain the observed transcriptional and stability changes in relation to other heterochromatin components characteristic of *Drosophila* telomeres, such as HP1a.

## Author Summary

*Drosophila* telomeres constitute a remarkable exception to the general telomerase mechanism of telomere maintenance in eukaryotes. The essential role of the telomeric transposons *HeT-A, TART*, and *TAHRE (HTT)* in this organism contrasts with the strong conservation of their retrotransposon *personalities*. The particularities of this system add an extra layer of complexity to the control of telomere length in *Drosophila*; on one hand, telomere expression should be fine-tuned in order to achieve telomere function whenever needed; on the other, terminal transposition should be tightly controlled to guarantee genomic stability. Here, we report the dual role of the JIL-1-Z4 complex in regulating the *HeT-A* retrotransposon transcription (by the action of the JIL-1 kinase) and in guaranteeing the stability of telomeres (by the zinc finger protein Z4). We show how the loss of JIL-1 and Z4 causes major changes at the chromatin of the *HeT-A* promoter that can explain the phenotypes that we observe in *JIL-1* and *Z4* mutant alleles. Moreover, we give evidence of the involvement of the HeT-A Gag protein in the recruitment of Z4 to the HTT array, and we demonstrate how the disruption of this interaction has fatal consequences for telomere stability.

## Introduction

Telomere elongation is needed in all eukaryotes with linear chromosomes due to the incapacity of cellular polymerases to proceed in 3′ to 5′ direction. Telomere length homeostasis is important for protecting the chromosomes from terminal erosion and the loss of important genetic information. Moreover, a defined telomere length is required for the proper assembly of the telomere-capping complex (*shelterin* in telomerase telomeres or *terminin* in *Drosophila*) [1]–[3]. When telomeres recess excessively, the disassembly of the protective cap leaves the telomere ends unprotected. Consequently, the telomeres are recognized by the DNA damage machinery, and upon repair are fused together resulting in genomic instability [4]. Eukaryote telomeres are dynamic structures that make up their telomere length from a balanced mechanism of gains and losses. The net result of this process is a telomere of the appropriate length to exert the different telomeric functions, as well as for protecting the genetic content [5], [6].

Several proteins have been described with both positive and negative effects on telomere length regulation [1]. Some of these cellular components act regulating the different telomerase subunits either by directly activating their expression, or their biochemical function [7]. On the other hand, changes on the telomeric chromatin have also been related to changes in telomere length in several organisms pointing to an epigenetic component in telomere regulation in eukaryotes [8]. Thus, telomere length homeostasis is a complex cellular process that integrates signals from different regulatory mechanisms.

*Drosophila* is the telomerase exception better studied so far, having acquired a retrotransposition based mechanism whose prevalence along all the genus (120 MY) demonstrates its robustness [6], [9], [10]. The success of this mechanism is based in the targeted transposition of three different specialized non-LTR retrotransposons, *HeT-A, TART* and *TAHRE* [11]–[14]. Retrotransposons belong to Class I transposable elements (TE), and their mechanism of transposition involves an RNA intermediate implying that each new successful transposition will increase the copy number of the element. This, in the case of the telomeric transposons will translate in increased telomere length directly benefiting the host and indirectly incrementing the absolute copy number of the telomeric transposons, ensuring their survival.

Recent molecular studies demonstrate that telomeres in most eukaryotes are composed of two domains; the protective cap that lies at the very end and the distal (telomeric) domain. Flanking the telomere domain lays the proximal (subtelomeric) domain, which shows different chromatin characteristics [8], [15], [16]. The telomeric domain is composed of the telomerase repeats in telomerase organisms and of the retrotransposon array *HeT-A*, *TART* and *TAHRE* (HTT) in the case of *Drosophila*. Andreyeva and collaborators took advantage of the *Drosophila melanogaster Telomere elongation* (*Tel*) mutant strain, which has telomeres ten times longer than the wild type in order to obtain a better resolution of the chromatin in the different telomeric domains [17]. Their study demonstrates that the HTT array shows mixed characteristics of euchromatin and heterochromatin, and that two chromosomal proteins, JIL-1 and Z4, specifically localize in this domain [15].

Z4, also known as Putzig, is a seven-zinc finger protein known to localize at polytene chromosome interbands and necessary to maintain the band-interband structure in these special chromosomes [18]. Moreover, Z4 has been shown to be an important cofactor in at least three different pathways related with chromatin remodeling: the NURF and the TRF2/DREF remodeling complexes, where it acts as an activator [19], [20]; and in the JAK/STAT pathway, where Z4 acts as a co-repressor [21]. With the exception of its role as a co-repressor in the JAK/STAT pathway, where Z4 binds to the Ken protein, Z4 exerts its effects mediating chromatin changes.

In *Drosophila*, JIL-1 is the chromosomal kinase in charge of the phosphorylation of Serine 10 of histone H3 during interphase [22]. Like Z4, JIL-1 also localizes at interbands in polytene chromosomes, and has a role in maintaining the band-interband structure of polytene chromosomes [23]. JIL-1 seems to have two interdependent functions; on one hand, JIL-1 is required for the structure of the chromosomes, and on the other, is involved in reinforcing active transcription of certain genes during interphase, as well as participating in the dosage compensation of genes in the male X chromosome [24]–[26]. Several JIL-1 interacting partners have been identified; JIL-1 interacts with Lamin Dm_0_, histone H3, and the chromosomal protein Chriz, also known as Chromator, among others [27]–[30]. A possible interaction between JIL-1 and Z4 has also been suggested [28].

Telomere function in *Drosophila* has been suggested to be epigenetically determined since the *terminin* complex that protects the telomeres assembles there in a sequence independent manner [2]. Maintenance of chromatin domains is a dynamic process in which chromatin marks and proteins interchange in a complex network of interactions. Each network, defines a set of characteristics that favor expression or repression of the genes embedded in such domains. Because telomere function in *Drosophila* strongly depends on transcription from the HTT array, where the genes involved in telomere elongation reside, the regulation of the chromatin structure of this domain is especially relevant to understand the regulation of telomere length in this organism. With the objective of better understanding this regulation, we have studied the role of JIL-1 and Z4, two chromosomal proteins shown to localize at the HTT array [15], on the regulation of the telomeric chromatin and their influence upon the expression of the main telomeric component in *D. melanogaster*, the *HeT-A* retroelement [31]. We have found that JIL-1 regulates *HeT-A* transcription, being thus the first described positive regulator of telomere expression in *Drosophila*. Moreover, we demonstrate that different Z4 mutant alleles show telomeric fusions in metaphase chromosomes from larval neuroblasts. ChIP experiments of *Z4* and *JIL-1* mutant alleles highlight changes of other components of the telomeric chromatin like the HP1a protein that can explain the transcriptional and stability effects observed in *JIL-1* and *Z4* mutant alleles. Importantly, we have also obtained proofs of a possible telomere targeting mechanism to recruit the JIL-1-Z4 complex to the telomeres.

## Results

In order to select candidate proteins that could have a role regulating the expression of the telomere retrotransposons, we took advantage of a study that characterizes the protein distribution along the different domains of *D. melanogaster* telomeres. Andreyeva and collaborators used the *Tel* stock, with telomeres ten times longer than in wild type flies, which offer a better resolution of the different telomeric domains [17], to perform immunocytochemistry experiments. These experiments demonstrated that the proteins JIL-1 and Z4 localize specifically at the HTT domain but neither at the capping nor at the TAS subtelomeric domain [15]. We have centered all analyses presented here on the *HeT-A* retrotransposon, which is the most abundant of the three telomeric retrotransposons in the HTT array of *D. melanogaster* [31].

### *HeT-A* transcription is regulated by JIL-1

**Figure 1:**
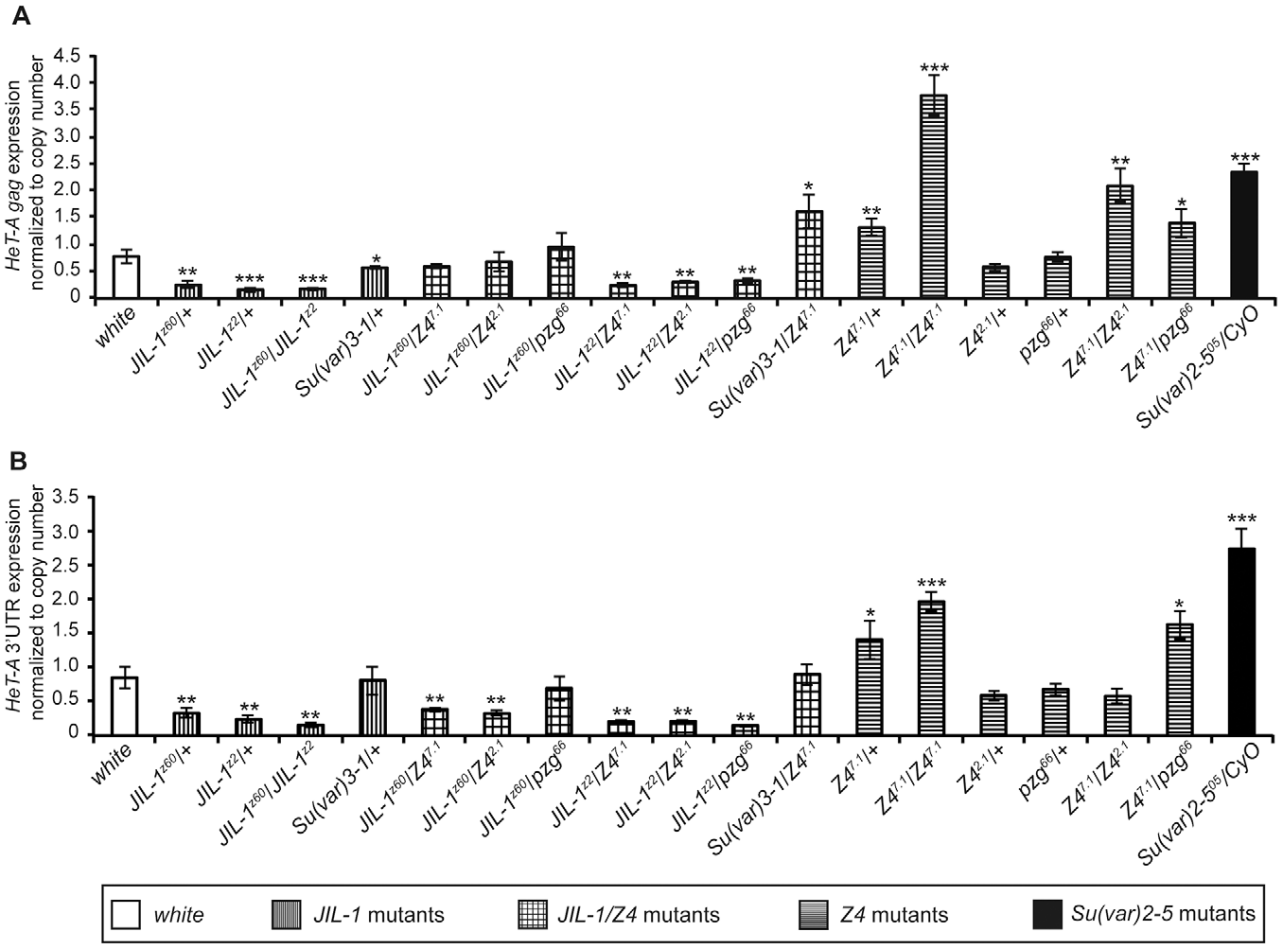
*HeT-A* expression normalized to copy number in different mutant alleles. *HeT-A Gag* (A) and *HeT-A* 3′UTR (B) transcripts decrease in *JIL-1* mutants (*JIL-1^z60^*/+, *JIL-1^z2^*/+ and *JIL-1^z60^*/*JIL-1^z2^*) and in *Z4* mutants when combined with *JIL-1^z2^* null allele (*JIL-1^z2^*/*z4^7.1^*, *JIL-1^z2^*/*Z4^2.1^* and *JIL-1^z2^*/*pzg^66^*). *Z4^7.1^* allele also affects *HeT-A Gag* transcripts but in this case an increase in the expression is observed. The *Su(var)2-5^05^* mutant allele, was used in this experiments as a positive control. *HeT-A* transcript levels were normalized to actin transcripts and corrected for the respective *HeT-A* copy number in each analyzed stock. Error bars represent standard deviations of three independent experiments. Asterisks indicate statistically significant differences using the t-test (one asterisk, *P*<0.05 to 0.01; two asterisks, *P*<0.01 to 0.001; three asterisks, *P*<0.001) in *HeT-A* expression of each mutant compared to *w^1118^*.

We analyzed the levels of mRNA of the *HeT-A* element in different *JIL-1* and *Z4* mutant alleles and, in order to contextualize our results, we compared them with the levels obtained from the mutant allele of one of the genes already known to influence *HeT-A* expression, the *Su(var)2-5* gene which encodes for HP1a (Figure 1A and 1B) [32], [33]. Different mutations in the *Su(var)2-5* gene, result in a pronounced increase in *HeT-A* transcription and severe problems of telomere stability [32], [33]. Because the number of *HeT-A* copies may vary among stocks, we determined the number of copies of the *HeT-A* element for each of the analyzed stocks (Figure S1A and S1B). We used this data to normalize the level of *HeT-A* transcription per number of copies and understand if *JIL-1* and *Z4* mutants show a differential *HeT-A* transcription activity (Figure S2A and S2B, Figure 1A and 1B). Because in any given stock full length and truncated *HeT-A* elements coexists at the telomeres [31], [34], we performed the quantitative Real-Time experiments with two different sets of primers separated more than 3 kb along the full length *HeT-A* transcript (*see materials and methods for primer details*).

To determine the level of *HeT-A* transcription we used whole third instar larvae to extract total mRNA. Some of the larval tissues in this stage, like the brain and imaginal discs, are in demand for active cell division and have been reported to show active *HeT-A* expression [35]. We analyzed a *JIL-1* hypomorph allele, *JIL-1^z60^* that contains a molecular lesion, which results in low levels of functional protein [22], and also a null allele *JIL-1^z2^* [25]. In addition, we analyzed the *JIL-1^Su(var)3-1^* allele, which has been suggested to be a gain of function allele [36]. Homozygous *JIL-1^z60^* animals have only a 17% of eclosion rate [22], and *JIL-1^z2^*/*JIL-1^z2^* are homozygous lethal [25].

We obtained a significant reduction of *HeT-A* transcription with both sets of primers (3′UTR and *gag* gene) compared to the control strain (*w^1118^*) for all the alleles with the exception of the *JIL-1^Su(var)3-1^* allele (Figure 1A and 1B, left section of the graphics). In this last case, we did not observe a decrease in *HeT-A* transcription probably due to the ectopic phosphorylation activity of JIL-1 in this allele [30]. The *JIL-1* alleles show telomere lengths comparable to wild type (Figure S1A and S1B). The three *JIL-1* alleles tested are in different genetic backgrounds, although for the hypomorph and the null mutations we have obtained very similar results, we crossed all the alleles with the *w^1118^* strain in order to minimize the contribution of the genetic background in these measures.

Similarly, we analyzed three different *Z4* mutant alleles, *Z4^7.1^*, *Z4^2.1^ and pzg^66^*. The *Z4^7.1^* allele is a hypomorph allele that lacks the promoter region of the *Z4* gene and is lethal at the pupal stage [18]. The *Z4^2.1^* and the *pzg^66^* alleles are null alleles that result in embryonic and early larval lethality [18], [21]. We obtained a substantial increase in *HeT-A* transcription in the case of the hypomorph allele *Z4^7.1^* (Figure 1A and 1B). The results are similar for both sets of primers used indicating that the increase affects most *HeT-A* copies. The increased *HeT-A* transcription in the *Z4^7.1^* allele is consistent with a major level of *HeT-A* transcripts in all the allelic combinations where this allele is present (Figure 1A and 1B). In addition, the number of copies of the *HeT-A* element in this allele was substantially increased indicating longer telomeres in this stock (Figure S1A and S1B). Note that the level of *HeT-A* transcription in the *Z4^7.1^*/*Z4^7.1^* homozygous is close to the *HeT-A* transcription in the *Su(var)2-5^05^* mutation of HP1a. The *Z4^2.1^* and *pzg^66^* alleles did not show a different level of *HeT-A* transcription compared to our control strain (*w^1118^*) for neither the 3′UTR region nor the *gag* gene. As expected the number of *HeT-A* copies in these null *Z4* alleles is not significantly different from the control strain (*w^1118^*). Only when the *Z4^7.1^* allele is combined with the *Z4^2.1^* or the *pzg^66^* alleles the levels of *HeT-A* transcription increase above the ones in the *w^1118^* strain. The levels of *HeT-A* expression concerning the *HeT-A gag* gene in the *Z4^7.1^/Z4^2.1^* and the *Z4^7.1^*/*pzg^66^* genotypes are significantly different from the ones obtained by the *Z4^7.1^*/*Z4^7.1^* homozygous combination. For the 3′UTR region, the difference in expression in only significant from the one obtained for the *Z4^7.1^*/*Z4^7.1^* genotype for the *Z4^7.1^/Z4^2.1^* allelic combination.

We also investigated if we could observe a genetic interaction between *JIL-1* and *Z4* by measuring the levels of *HeT-A* transcription of a total of seven allelic combinations between *JIL-1* and *Z4* (Figure 1A and 1B, Figure S2A and S2B). Although for the *Z4^2.1^* and *pzg^66^* alleles we had not detected levels of *HeT-A* transcription significantly above the ones of *w^1118^*, we found that the combinations *JIL-1^z60^/Z4^7.1^* and *JIL-1^z60^/Z4^2.1^* for the *gag* gene, and the *JIL-1^z60^/pzg^66^* for both the 3′UTR region and the *gag* gene recover *HeT-A* transcription to *w^1118^* levels (Figure 1A and 1B). Accordingly with the results obtained for the single mutation, in the combination *JIL-1^Su(var)3-1^*/*Z4^7.1^* we obtained levels of *HeT-A gag* transcription above the ones in *w^1118^*.

### JIL-1 regulates transcription by chromatin changes in the *HeT-A* promoter

**Figure 2:**
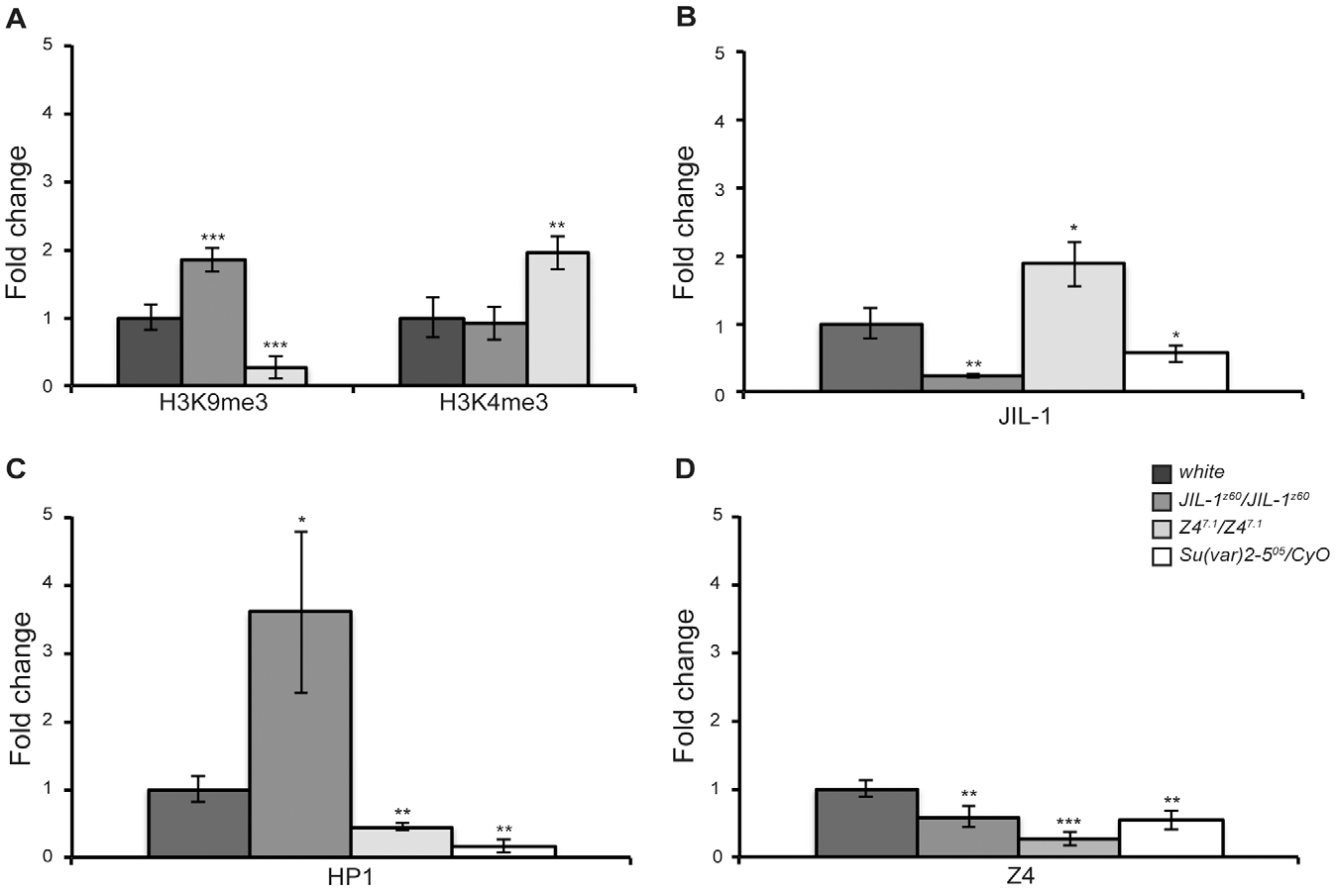
Chromatin changes at the *HeT-A* promoter. Chromatin Immunoprecipitation (ChIP) analyses were performed at the *HeT-A* promoter of *JIL-1*, *Z4* and *Su(var)2-5* mutants. Specific antibodies were used against H3K4me3 and H3K9me3 (A), JIL-1 (B), HP1 (C) and Z4 (D). In (A) significant changes are observed at H3K9me3 in both *JIL-1* and *Z4* mutants, while H3K4me3 only varies in *Z4* mutants. In (B), *Z4* mutants show an increase in JIL-1 protein at *HeT-A* promoter. In (C) HP1 occupancy increases in *JIL-1* and decreases in *Z4* mutants. (D) Both *JIL-1* and *Su(var)2-5* mutants show a decrease in Z4 levels at *HeT-A* promoter. Graphs represent ChIP quantitation using real-time PCR. Each sample was normalized to 10% input DNA, to RpL32 locus and to the respective control. Error bars represent standard deviations of three independent experiments. Asterisks indicate statistically significant differences (one asterisk, *P*<0.05 to 0.01; two asterisks, *P*<0.01 to 0.001; three asterisks, *P*<0.001) between mutant and *w^1118^* strains.

Because both JIL-1 and Z4 are proteins related with chromosome structure, we studied if the changes observed in the expression of the *HeT-A* retrotransposon were related to changes in the chromatin environment at the promoter of this retroelement. Therefore, we investigated by Chromatin immunoprecipitation (ChIP) experiments, changes in different chromatin marks and changes in the levels of the proteins of study, JIL-1 and Z4 (Figure 2), as well as of HP1a, a protein already known to localize at the HTT array and to affect *HeT-A* transcription (Figure 1A and 1C), [15], [16], [33].

To analyze the relative chromatin changes in the homozygous mutant alleles *JIL-1^z60/^JIL-1^z60^* and in the hypomorph *Z4^7.1^/Z4^7.1^* allele which had been the only one with increased *HeT-A* transcription, we measured trimethylation of both lysine 9 and 4 of Histone H3 (H3K9me3 and H3K4me3), the most characteristic histone modifications indicative of repressed and active chromatin. Figure 2A shows the relative changes, compared to wild type, in homozygous mutant alleles of *JIL-1* (*JIL-1^z60^/JIL-1^z60^)*, and *Z4* (*Z4^7.1^/Z4^7.1^)*. The increase observed for H3K9me3 in *JIL-1^z60^/JIL-1^z60^* mutants is in accordance with the decrease of *HeT-A* expression in the same allelic combination. In contrast, the increase in *HeT-A* transcription of the *Z4^7.1^/Z4^7.1^* allele has two different causes, a substantial decrease in H3K9me3 and a simultaneous increase in H3K4me3, indicative of active transcription.

Next, we quantified the presence of JIL-1 at the *HeT-A* promoter in mutant and wild type flies. Figure 2B shows the changes in JIL-1 occupancy at the *HeT-A* promoter. *Z4^7.1^/Z4^7.1^* mutant flies show an increase in JIL-1, in accordance with a higher expression of *HeT-A* in this mutant allele. In contrast, *Su(var)2-5^05^/CyO* flies, heterozygous mutant for HP1a, show a moderate decrease of JIL-1 occupancy suggesting a subtle dependence between these two proteins. Interestingly, *JIL-1^z60^/JIL-1^z60^* shows a substantial increase in the presence of HP1a at the *HeT-A* promoter, which could be in part responsible for the silencing of *HeT-A* expression in this same allele (Figure 2C). The *Z4^7.1^/Z4^7.1^* allele on the other hand, shows HP1a levels comparable to *Su(var)2-5^05^/CyO* flies suggesting an interdependent relationship between these two chromosomal proteins. Finally, we observed that the presence of the Z4 protein decreases in *JIL-1* and *Su(var)2-5* mutants further interconnecting these three chromosomal proteins in their role of chromatin modulators at the *HeT-A* promoter (Figure 2D).

We did not perform ChIP analyses in the case of the *Z4^2.1^* and *pzg^66^* alleles for the *HeT-A* promoter because of two reasons; these alleles did not show a significant difference in *HeT-A* transcription, very few third instar larvae of the *pzg^66^/Z4^7.1^* and *Z4^2.1^/Z4^7^*.^1^ genotypes are obtained from each cross, and for the combination *pzg^66^/Z4^2.1^* no animals eclose, making very difficult to perform this experiment with the adequate amount of material.

### JIL-1 interacts with Z4

**Figure 3:**
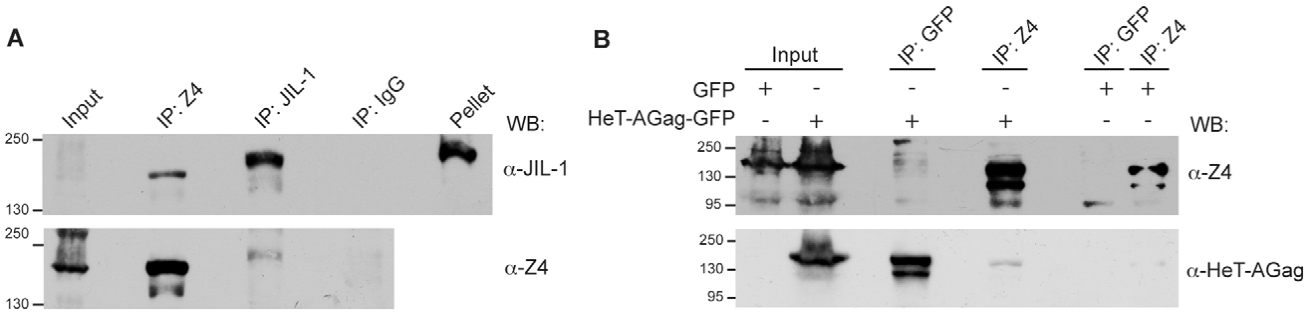
Z4 interacts with JIL-1 and *HeT-A* GAG. (A) Z4 and JIL-1 immunoprecipitation was performed in S2 cells using anti-JIL-1 and anti-Z4 antibodies. Negative control experiments were performed by immunoprecipitating with unspecific IgGs. (B) Z4 and *HeT-A* GAG immunoprecipitation was done by transfecting S2 cells with HeT-A Gag-GFP and immunoprecipitating with αnti-GFP and αnti-Z4. Control experiments were performed by transfecting an empty GFP vector (pPL17). Presence of the recombinant protein is indicated on the top of the panel (+ and − symbols). Antibodies used for immunoprecipitation are indicated on the top. All extracts were fractionated by SDS-PAGE, western blotted, and developed with specific antibodies (indicated on the right of each figure). Molecular markers (kDa) are indicated on the left.

Because JIL-1 and Z4 localize similarly in polytene chromosomes and in the HTT array [15], [18], [22], [23], and both of them had been found to directly interact with Chromator [27], [28], we wondered if the two proteins could be directly or indirectly interacting. We thus performed a co-immunoprecipitation experiment with the endogenous proteins. In Figure 3A we show how both proteins JIL-1 and Z4 are able to co-immunoprecipitate in Schneider S2 cells. Although the input lane of JIL-1 shows a very faint signal caused by the fact that a considerable amount of protein is not extracted and remains in the cell pellet, the protein is clearly detectable in the IP with the Z4 antibody, thus suggesting that a significative amount of soluble or extractable JIL-1 is part of a complex containing Z4. In the case of Z4, no substantial amount of the protein was detected in the pellet (*not shown*). The results of the co-immunoprecipitation experiments suggest that Z4 and JIL-1 belong to the same protein complex when they are at different genomic locations, such as at the HTT array.

### Z4 interacts with HeT-A Gag

JIL-1 and Z4 specifically localize at the HTT array but not at the TAS or the cap domain, therefore a specific telomere targeting mechanism should be in place. One of the proteins that specifically localizes at *Drosophila* telomeres, is the Gag protein of the *HeT-A* element [37], [38]. We therefore tested if HeT-A Gag could be involved in the targeting mechanism of Z4 and JIL-1 to the HTT array. We set a co-immunoprecipitation experiment with a recombinant form of HeT-A Gag fused to GFP together with the endogenous Z4 protein. Figure 3B shows that the HeT-A Gag-GFP protein co-immunoprecipitated with Z4, and that conversely Z4 co-immunoprecipitated with HeT-A Gag-GFP. Although we have not been able to detect co-immunoprecipitation of the endogenous HeT-A Gag with Z4 (we assume that due to low levels of expression of HeT-A Gag in most *Drosophila* tissues and cells), the overall data suggest that the Z4-HeT-A Gag interaction likely occurs *in vivo*. We did not detect a JIL-1-HeT-A Gag interaction (*data not shown*).

### *Z4* and *HeT-A gag* mutants show telomere instability in mitotic cells revealed by the appearance of telomeric fusions

Because changes in telomere length and telomere chromatin can result in telomere instability, we checked whether *JIL-1* and *Z4* mutant alleles showed any sign of genomic instability detectable by telomere fusions (TFs). We checked metaphase chromosome preparations from third instar larvae neuroblasts of *JIL-1* and *Z4* mutants and compared them to a negative control (*w^1118^*) and positive controls (mutant alleles of genes known to participate in telomere protection in *Drosophila)* like *woc* and *caravaggio*, the gene encoding the HOAP protein [39], [40].

**Figure 4:**
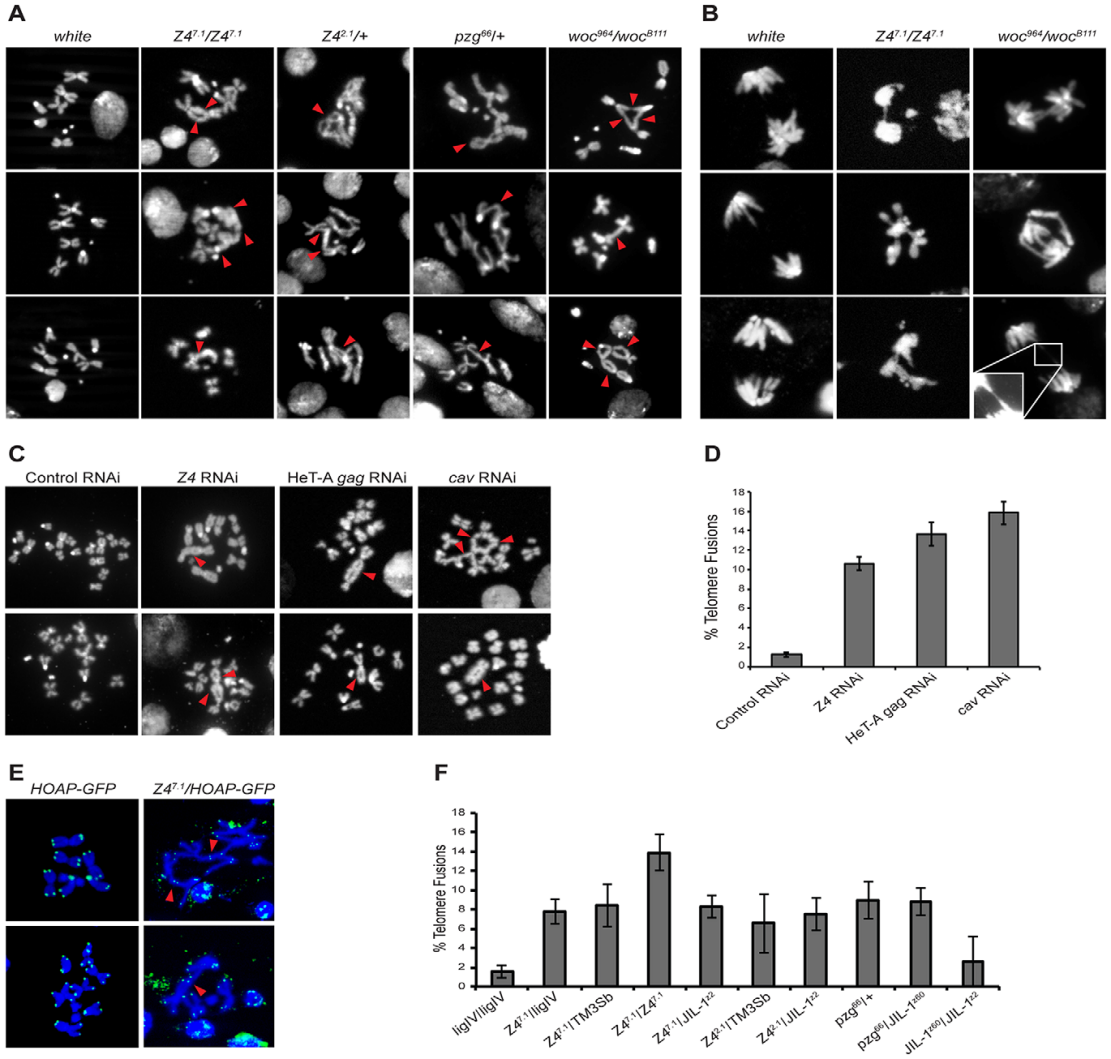
Telomere fusions in *Z4* and *HeT-A* mutants. (A) Mitotic chromosome preparations of third instar larvae. *Z4^7.1^/Z4^7.1^*, *Z4^2.1^*/+ and *pzg^66^*/+ alleles show telomere fusions. *woc^964/^woc^B111^* mutants were used as a positive control and *w^1118^* as negative control. Red arrowheads indicate telomere fusions. (B) Anaphase preparations from third instar larvae neuroblasts of the *Z4^7.1^/Z4^7.1^*, *woc^964/^woc^B111^* alleles and *w^1118^.* Defective anaphases due to telomere fusions visible in *Z4^7.1^/Z4^7.1^*, *woc^964/^woc^B111^* (positive control). Normal anaphases in *w^1118^* (negative control). (C) Mitotic S2 cells stained with DAPI after *Z4*, *HeT-A gag* gene, *cav* (HOAP), and unspecific (*Sart1*) RNAi treatment. Telomere fusions are observed in *Z4* and *HeT-A Gag* RNAi mutants; RNAi for the *cav* and the *Sart1* non-LTR retrotransposon from *Bombyx mori* were used as positive and negative control respectively. Red arrowheads indicate telomere fusions. (D) Percentage of telomere fusions found in mitotic S2 cells after RNAi treatment. (E) Mitotic chromosome preparations of HOAP-GFP/HOAP-GFP and *Z4^7.1^*/HOAP-GFP third instar larvae neuroblasts. Chromosomes stained with DAPI (blue) and HOAP-GFP fusion protein (green). HOAP is present in *Z4* telomere fusions (red arrowheads). (F) Percentage of telomere fusions found in *Z4^7.1^/ligIV* and *Z4*/*JIL-1* double mutants. *ligIV* and *JIL-1* mutants do not affect the number of telomere fusions in *Z4* mutants. For each mutant a minimum of 100 metaphases/anaphases of three different preparations were analyzed.

We could observe TFs involving the same chromosome (intra-chromosomal) and different chromosomes (inter-chromosomal) in all the *Z4* mutant alleles present in this study *Z4^7.1^, Z4^2.1^* and *pzg^66^*, (Figure 4A, 2^ond^, 3^rd^ and 4^th^ column). Similarly, TFs were observed in neuroblasts from the positive control, *woc^964^/woc^B111^* mutant allele (Figure 4A 5^th^ column). No TFs were observed in neuroblast preparations of the negative control stock (*w^1118^*) (Figure 4A 1^st^ column).

We further investigated whether the observed TFs in the *Z4* mutant alleles could be resolved during the next anaphase with no other consequences for the cell, or in contrast could cause asymmetric heredity of the genomic content and initiate genomic instability. We analyzed anaphase neuroblasts of *Z4^7.1^/Z4^7.1^* third instar larvae and compared them again with a positive (*woc^964^/woc^B111^*) and a negative control (*w^1118^*). Figure 4B (2^nd^ column) shows different anaphases of the *Z4^7.1^/Z4^7.1^* neuroblasts where chromatin bridges (1^st^ and 3^rd^ panel), and aberrant DNA content (2^nd^ and 3^rd^ panels) can be observed. Similarly, different chromatin bridges were observed for neuroblast preparations of *woc^964^/woc^B111^* larval brains (3^rd^ column Figure 4B). No abnormal anaphases were observed for the *w^1118^* neuroblast preparations (1^st^ column Figure 4B).

In order to rule out a possible unrelated effect of the genetic background in the *Z4* mutant alleles, we knocked down *Z4* by RNA interference in S2 cells. Intra and inter-chromosomal TFs were also detected after the preparation of metaphase chromosomes of the interfered cells for the *Z4* gene (Figure 4C, 2^nd^ column and Figure 4D). Again TFs were detected in the positive control (S2 cells interfered for the *caravaggio* gene, encoding the HOAP protein) (Figure 4C 4^th^ column and Figure 4D) and no TFs were observed when the S2 cells were interfered for an unrelated RNA (Figure 4C 1^st^ column, and Figure 4D, *see materials and methods for details*).

As we had observed an interaction between Z4 and HeT-A Gag, we decided to test if the lack of the latter could also result in telomere instability. Due to the impossibility to obtain mutant alleles for *HeT-A* in *D. melanogaster* (many copies of the *HeT-A* element exist in any given stock, [31]), we decided to interfere for the *HeT-A* retrotransposon mRNA in S2 cells by RNAi. Figure 4C, 3^rd^ column and Figure 4D show how a decrease on *HeT-A* mRNA and, as a consequence, on the levels of HeT-A Gag protein results in different TFs in metaphase chromosomes, involving chromatids from the same chromosome and from different chromosomes. Obtaining similar TF phenotypes when interfering for HeT-A Gag and Z4 reinforces the relationship of these two proteins at the HTT array.

In order to investigate other possible causes for the telomeric instability observed in the *Z4* mutant alleles, apart from the changes in the telomeric chromatin in these mutants, we tested two alternative hypothesis; 1) disturbance of the loading of the telomere-capping complex and 2) the possible involvement of the non-homologous end joining DNA repair complex in fusing the telomeres after being recognized as a double strand break by the non-homologous end-joining pathway [41]. Thus, we investigated if the loading of one of the major capping components, the HOAP protein [40], was perturbed in *Z4* mutants by crossing them with flies with an endogenous HOAP-GFP protein. In Figure 4D (2^nd^ column), HOAP signals can be distinguished in TFs from metaphase chromosomes of the *Z4^7.1^/HOAP-GFP* allele suggesting that at least part of the capping complex is still able to recognize and be loaded at the telomere (see arrowheads for HOAP signals over different TFs, Figure 4D). No TFs are seen in the HOAP-GFP metaphase chromosomes (Figure 4D 1^st^ column). In order to rule out a possible contribution of the non-homologous end joining repair complex, we analyzed the contribution of the Ligase IV enzyme in the observed TFs in the *Z4* mutant alleles [41], by combining the *Z4* mutation (*Z4^7.1^*) with a mutation for the gene encoding the Ligase IV enzyme (*ligIV^−/−^*). Therefore, in case that the TFs observed in the *Z4* mutants were caused by this mechanism, we should have seen a decrease in the number of TFs when the two mutations are combined. The *ligase IV* allele that we assayed, (*ligIV^−/−^*) does not show a TF phenotype compared with our control strain, (*w^1118^*) (Figure 4F). As shown in Figure 4E, the TF number detected in *Z4^7.1^/ligIV* double mutant was not statistically different from the *Z4^7.1^/TM3Sb* allele. The results from these experiments strongly suggest that Z4 controls telomere stability independently of the DNA repair machinery and that Z4 is not directly involved in the loading of the telomere-capping complex.

We also investigated if the mutant alleles for *JIL-1* might also have a problem of telomere stability. We inspected metaphase chromosomes of 3^rd^ instar larva neuroblasts for the *JIL-1^z60^, JIL-1^z2^* and *JIL-1^Su(var)3-1^*mutant alleles and did not find any significant telomere fusion (TFs) phenotype compared to *w^1118^* (*data not shown*). We also inspected the possibility of TFs in the trans-heterozygous combination *JIL-1^z60^/JIL-1^z2^* and found no result significantly different from the *w^1118^* strain (Figure 4F).

**Figure 5:**
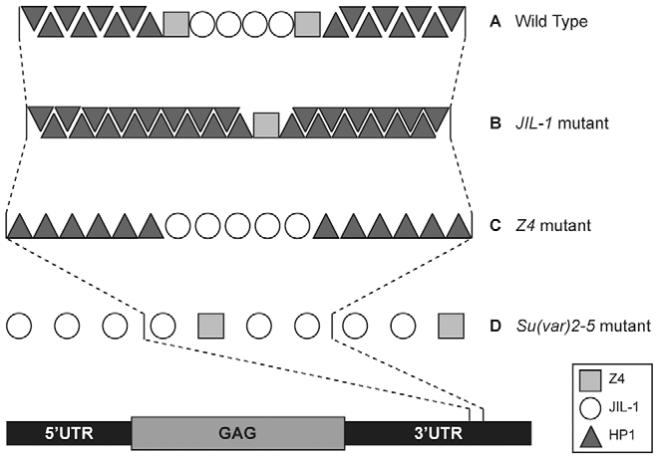
Model of the chromatin environment at the *HeT-A* promoter. (A) Wild type: Z4 defines a boundary at *HeT-A* promoter that protects from the action of HP1a and other heterochromatin markers. JIL-1 guarantees a certain level of euchromatin inside the *HeT-A* promoter in order to allow gene expression. (B) *JIL-1* mutants: destabilization of the Z4 boundary and the heterochromatin spreads into the *HeT-A* promoter (enrichment in HP1 and H3K9me3). (C) *Z4* mutants: Disappearance of the Z4 boundary, increase in euchromatin marks (H3K4me3) and decrease in heterochromatin marks (HP1a and H3K9me3). Subtle increase in JIL-1 and in euchromatinization of the *HeT-A* promoter. (D) In *Su(var)2-5* mutants: The lack of HP1a allows relaxation of the Z4 boundary causing a JIL-1 and Z4 spread along the HTT array and a relative decrease of these proteins inside the *HeT-A* promoter. Although the levels of JIL-1 inside the *HeT-A* promoter are lower than in wild type, the release of silencing caused by loss of HP1a results in increased *HeT-A* expression.

Next, we decided to test if a *JIL-1* mutant in a *Z4* mutant background could rescue the TF phenotype. Because in a *JIL-1* mutant background some heterochromatin marks increase their presence in the *HeT-A* promoter, (H3K9me3 and HP1a, Figure 2A and 2C and Figure 5) it is possible that they are enough to compensate the lower amount of Z4 in the *JIL-1* mutation (Figure 2D). With this purpose we tested the double mutant combinations (*Z4^7.1^/JIL-1^z2^, pzg^66^/JIL-1^z60^*, *z4^2.1^/JIL-1^z2^*) and we found no significant difference between the single *Z4* mutant alleles (Figure 4F), indicating that the partial increase in heterochromatin marks is not sufficient to compensate the lower levels of Z4 in a *JIL-1/Z4* mutant background. Therefore, the role of Z4 in the structure of the telomere chromatin is key to guarantee telomere stability in *Drosophila*.

## Discussion

### JIL-1 is the first positive regulator of telomeric expression described in *Drosophila*

Much effort has been put forward to study the negative regulation of the telomeric retrotransposons [42] (for a review, see [6]) as these elements have been able to maintain their *personalities* or individual characteristics as transposable elements while fulfilling a cellular role [9], [43]–[45].

*HeT-A* is a retrotransposon with the essential function of telomere elongation, and therefore a fine-tuned regulation capable of achieving both, telomere replication and avoiding putative harmful transpositions and consequently genomic instability should be in place to ensure a normal telomere structure. During development, all the tissues that undergo active cell division such as the brain or the imaginal discs need certain levels of telomere replication. Naturally, these are the tissues where the telomeric retrotransposons are more expressed [35]. Here, we demonstrate that the JIL-1 kinase is important to achieve wild type levels of *HeT-A* transcription in larval tissues, being the first positive regulator of telomere transcription described in *Drosophila*.

Although in *Drosophila* the role of JIL-1 in activating transcription has remained controversial [46], [47], at least in the HTT array it could act as a positive regulator of transcription for three different reasons: 1) When telomere elongation is needed, a fast activation of *HeT-A* transcription should be expected. Accordingly, the mammalian *JIL-1* orthologous MSK1/2 have been shown to rapidly induce gene expression on the face of stress or steroid response [48]. 2) *HeT-A* is embedded into the HTT array, a domain that needs to be protected from the influence of the repressive heterochromatin of the neighboring TAS domain [16]. JIL-1 has been suggested to protect the open chromatin state from the spreading of neighboring repressive chromatin at certain genomic positions [49], [50]. 3) The decrease in expression that we have observed in the *JIL-1* mutants is moderate (Figure 1C). Recent data at genomic level revealed that JIL-1 function agrees with a reinforcement of the transcriptional capability of a particular genomic domain rather than net activation [51]. In summary, the telomeric role of JIL-1 at the HTT array is in agreement with all of the above.

Phalke and co-workers [52] suggest that JIL-1 has a role in retrotransposon silencing in general and has no effect on telomere transcription. A possible explanation for this discordance with our results and hypothesis is that the mutant allele of JIL-1 assayed by Phalke and co-workers, the *JIL-1^Su(var)3-1^* allele, corresponds to a C-terminal deletion of the JIL-1 protein that causes the protein to miss-localize and phosphorylate ectopic sites [30], [36]. The ectopic phosphorylation caused by the *JIL-1^Su(var)3-1^* allele would activate the expression above wild type levels in those genes that normally are not targeted by JIL-1, as it happens to be the case for the *Invader4* retrotransposon. In our study, we have assayed the *JIL-1^Su(var)3-1^* allele obtaining similar result than for the wild type stock, likely for similar reasons (Figure 1A and 1B). Supporting this, in addition of the *JIL-1^Su(var)3-1^*, we present here data from two more *JIL-1* alleles (Figure 1), *JIL-1^z60^ and JIL-1^z2^*, that correspond to loss of function alleles and, in both cases, result in a substantial decrease in *HeT-A* transcription (Figure 1A and 1B). Moreover, the changes in telomere transcription that we report here have been assayed directly on the major component of the HTT array, and not through a reporter [52]. Our data demonstrates that JIL-1 is necessary to maintain active transcription of the telomeric retrotransposon *HeT-A* or, what is the same, transcription from the telomeres in *Drosophila*.

Although we have demonstrated that JIL-1 is necessary to maintain transcription from the HTT array, we have not detected a decrease in telomere length in the *JIL-1* mutant alleles. A reasonable explanation for this observation is that the *JIL-1* mutant alleles here analyzed (*JIL-1^z60^* and *JIL-1^z2^*) have been maintained as heterozygous. It is therefore possible that one copy of *JIL-1* is enough to promote enough *HeT-A* transcription to elongate significantly the telomeres when needed.

### Z4 is necessary to guarantee telomere stability in *Drosophila*

Although in the case of the hypomorph mutation *Z4^7.1^* we have observed an increase in *HeT-A* transcription and *HeT-A* copy number significantly above the control strain (*w^1118^*), the null alleles *Z4^2.1^* and *pzg^66^* do not show an up-regulation of *HeT-A* transcription or an increase in its copy number (Figure 1A and 1B, Figures S1 and S2). Although we have crossed all the stocks to the *w^1118^* strain to minimize the effects of the genetic background, it could still have a certain influence when comparing the *pzg^66^* allele with the *Z4^7.1^*. Nevertheless the *Z4^7.1^* and *Z4^2.1^* alleles come from the same genetic background [18]. A possible explanation could rely on the fact that the *Z4^7.1^* mutation is a hypomorph mutation where a small amount of Z4 protein is still present. By ChIP analyses we have detected an increase of JIL-1 protein at the *HeT-A* promoter above control levels, which could explain in part the major transcription of *HeT-A* in this mutant background (Figure 2B). Because Z4 and JIL-1 interact (Figure 3A), it is possible that although low, the amount of Z4 present in the *Z4^7.1^* allele is enough to recruit JIL-1 to the *HeT-A* promoter. In the *pzg^66^* and the *Z4^2.1^* null alleles, JIL-1 cannot be recruited towards the *HeT-A* promoter and there is no increase in transcription. Nevertheless, with our current data we cannot conclude that Z4 directly controls the level of *HeT-A* transcription.

We have detected a phenotype of telomere instability in all three *Z4* mutant alleles *Z4^7.1^, Z4^2.1^* and *pzg^66^* (Figure 4), suggesting a role of this chromosomal protein in guaranteeing telomere stability in *Drosophila*. Although a number of genes involved in the capping function in *Drosophila* still remain unidentified [3], we do not have evidences that Z4 directly participates in the protection of the telomeres. Mutant alleles of genes directly involved in the capping function, such as *woc* or *caravaggio* (HOAP), show multiple and more numerous TFs in larval neuroblasts (Figure 4A, 4C and [39], [40]) than the ones that we have observed in the *Z4* mutant alleles. Moreover, we have been able to detect staining for one of the major capping components, the HOAP protein, in the TFs of *Z4* mutant neuroblast cells (Figure 4D), indicating that the telomere-capping complex is still loaded to a certain degree. Instead of directly participating in the capping, our hypothesis is that the major chromatin changes caused by the lack of Z4 at the HTT array result in a secondary loss of necessary chromatin and capping components like HP1a (Figure 2C).

Results from the ChIP experiments (Figure 2) suggest a relationship between JIL-1, Z4 and HP1a in fine-tuning the chromatin structure at the HTT array. HP1a has a dual role at the telomeres explained by its participation in both the capping function and the repression of gene expression that also exerts in other genomic domains [33], [53]. In the HP1a *Su(var)2-5^05^* allele, which it is known to have a major transcription of *HeT-A* and problems of telomere stability, we have observed a pronounced decrease in Z4 and JIL-1 (Figure 2B and 2D). In the *Z4^7.1^* allele the decrease in Z4 protein is accompanied by a similar decrease in H3K9me3 and HP1a at the *HeT-A* promoter (Figure 2A, 2C and 2D). Finally in the *JIL-1^z60^* allele the increase in silencing epigenetic marks like H3K9me3 and HP1a is also accompanied by a decrease in Z4 (Figure 2). In particular, the pronounced dependence of the presence of HP1a and Z4, points toward the loss of HP1a and H3K9me3 to a possible cause for telomere instability in the *Z4* mutant alleles here studied. Interestingly, in the *Su(var)2-5^04^/Su(var)2-5^05^* heteroallelic combination (considered a null mutation) [33]), 15% of telomeres involved in telomere associations are still able to recruit the HOAP protein [40]. Therefore our data on HOAP localization in the *Z4* mutant alleles is still consistent with the TFs being caused by the decreased availability of HP1a in these cells (Figure 2C). The above results demonstrate that Z4 in a coordinated manner together with JIL-1 and HP1a is an important component of the telomere chromatin in *Drosophila*, which upon its reduction causes significant changes in the chromatin of the HTT array, which are the cause of the observed telomere instability in all the *Z4* mutant alleles here studied (*discussed below*, Figure 5).

### HeT-A Gag targets the Z4-JIL-1 complex to the HTT array

We have been able to detect a biochemical interaction between JIL-1 and Z4, and our data suggests that these two proteins can be components of the same protein complex (Figure 3A). This interaction had been previously suggested because both proteins have been found co-localizing in different genomic locations, but no direct proof existed to date [15], [18], [22], [23], [27], [28]. In each genomic location where the Z4-JIL-1 complex is needed, a special mechanism of recruitment should exist. Importantly, we have shown how Z4 specifically interacts with HeT-A Gag (Figure 3B). HeT-A Gag is the only protein encoded by the *HeT-A* element and has been shown to specifically localize at the telomeres [37], [38]. HeT-A Gag has been shown to be in charge of the targeting of the transposition intermediates for the *HeT-A* element and also for its telomeric partner the *TART* retrotransposon [37]. Interestingly, when we studied the consequences for telomere stability after knocking down the *HeT-A gag* gene by RNAi, we also observed similar TFs than when knocking down the *Z4* gene, further relating the action of both genes in telomere stability. Z4 is known to participate in different protein complexes with roles in different genomic locations [19], [21], [27]. Because it has been demonstrated that Z4 is able to associate with a variety of proteins in these complexes, we think that the description of a mechanism for its specific targeting to telomeres through one of the telomeric retrotransposon proteins is especially relevant.

### A model for the role of JIL-1 and Z4 in *Drosophila* telomeric chromatin

Integrating information from previous literature and the results exposed by this study, we propose a possible model to describe the state of the chromatin at the HTT array in each of these three mutant scenarios; *JIL-1*, *Z4* and *Su(var)2-5*, as well as in *wild type* (Figure 5).

We should take into account that 1) HP1a has been shown to spread along the *HeT-A* sequence [16]. 2) The structure and the phenotypes of the different *Z4* mutant alleles suggest a possible role of this protein in setting and maintaining the boundaries between heterochromatin and euchromatin in polytene chromosomes [18], [28]. 3) JIL-1 has been extensively shown to be important to counteract heterochromatinization and, when missing, causes a spreading of heterochromatin markers such as H3K9me2, HP1a and Su(var)3-7 [49], [50], [54], [55]. 4) JIL-1 has been found to co-localize with Z4 at the band-inter-band transition in polytene chromosomes and also to co-purify with Z4 in different protein complexes [18], [27], [28]. In addition to this, we have been able to detect a biochemical interaction between JIL-1 and Z4 (Figure 3A), as well as, a certain dependence on the presence of JIL-1 for the proper localization of Z4 (Figure 2D), suggesting a possible role of JIL-1 upstream of Z4. Finally, 5) The ChIP analyses in this study suggest a certain dependence of Z4 on HP1a or onto similar chromatin requirements for the loading of both proteins at the HTT array, more specifically at the *HeT-A* promoter (Figure 2A, 2C and 2D). Summarizing all of the above, we propose that the chromatin at the *HeT-A* promoter could have the following structure:

In a *wild type* situation (Figure 5A), the *HeT-A* promoter contains intermediate levels of HP1a, JIL-1 and Z4. HP1a would be spread along the HTT array, JIL-1 would be concentrated at the promoter region of *HeT-A* guaranteeing certain level of expression and Z4 would be important to set the boundary between these two opposite modulators.

In a *JIL-1* mutant, (Figure 5B), the lack of JIL-1 would disturb the Z4 boundary causing a slight decrease in the Z4 presence. This result is in agreement with a Z4-JIL-1 partial interaction (Figure 3A and [28]). The decrease in JIL-1 presence and the disturbance of the boundary causes a spreading of HP1a into the *HeT-A* promoter, increasing its presence and repressing transcription from the HTT array (Figure 1 and Figure 2).

In a *Z4* mutant (Figure 5C), the disappearance of the boundary together with the significant decrease in H3K9me3 causes a decrease in HP1a binding and a substantial modification of the chromatin at the HTT array (Figure 2). The lack of sufficient HP1a at the HTT array causes a destabilization of the chromatin at the *cap* domain triggering telomere instability as a result (Figure 4). This scenario applies to the three *Z4* mutant alleles present in this study, the hypomorph *Z4^7.1^*, and the nulls *pzg^66^* and *Z4^2.1^*. On one hand the loss of some Z4 in *Z4^7.1^*/*Z4^7.1^* genotype produces overexpression of *HeT-A* because in addition to a relaxation of the chromatin, part of JIL-1 is still recruited to the *HeT-A* promoter (Figure 2A and 2B) and activates transcription in a more effective way than in a wild type situation.

Finally, in a *Su(var)2-5* mutant background (Figure 5D), the lack of HP1a along the *HeT-A* sequence allows a relaxation of the boundary causing a spread of JIL-1 and Z4 from the *HeT-A* promoter towards the rest of the array and creating as a consequence, permissive chromatin environment releasing *HeT-A* silencing (Figure 1, Figure 2, and [33]).

Our model does not completely explain the complex relationships that regulate telomere chromatin, likely because other important components are yet to be described or associated with the ones presented here. For example, other chromatin regulatory components that have been associated with *Drosophila* telomeres are such as: the deacetylase Rpd3, with a regulatory role on chromatin structure [56], and the histone methyltransferase SetDB1 and the DNA methylase Dnmt2 [52], [57] which by acting in the same epigenetic pathway repress transcription of *HeT-A* as well as of retroelements in general [52]. Future in depth studies on additional chromatin components will allow us to complete and detail even more the description of the chromatin at the HTT array, and allow a better understanding of the mechanism of retrotransposon telomere maintenance and the epigenetic regulation of eukaryote telomeres in general. In the meantime, here we describe a plausible scenario in the view of our transcription and ChIP data.

The results shown here demonstrate the role of JIL-1 as the first described positive regulator of telomere (i.e. *HeT-A*) expression in *Drosophila*. Because *HeT-A* is in charge of telomere maintenance in *Drosophila*, these results are key to understand how telomere elongation is achieved in retrotransposon telomeres. We also demonstrate that Z4 is necessary to guarantee telomere stability. The data presented here strongly suggest that JIL-1 and Z4 exert these functions by maintaining an appropriate telomere chromatin structure by a coordinated action together with other known telomere components such as HP1a. Moreover, we show that JIL-1 and Z4 interact biochemically. Last, and importantly for understanding how the specific role of the Z4-JIL-1 complex at the telomeres is defined and differentiated from its role in other genomic regions, we show that Z4 might interact with the HeT-A Gag protein, providing evidence for a targeting mechanism that specifically retrieves this complex to the telomeres.

## Materials and Methods

### Fly stocks and crosses

Fly stocks were maintained and crosses performed at 25°C on standard *Drosophila* corn meal medium. *w^1118^* strain was used as control. *JIL-1^z60^/TM6*, *JIL-1^z2^/TM6* and *JIL-1^Su(var)3-1^/TM3SbTb* stocks were provided by Kristin M. Johansen. *Z4^7.1^/TM3Sb* and *Z4^2.1^/TM3Sb* came from Harald Eggert and Harald Saumweber. *pzg^66^*/TM6 from [21] was a kind gift of Anja Nagel. The stocks *ligIV ^−/−^* and HOAP-GFP were obtained from Yikang Rong. The *woc^964^/TM6* and *woc^B111^/TM6* alleles were provided by Maurizio Gatti. *Su(var)2-5^05^/CyO* was obtained from Bloomington Stock Center.

### Genomic DNA extraction

Genomic DNA was extracted from adult flies to quantify the number of *HeT-A* copies in each strain. Ten third instar larvae without salivary glands were homogenized in 200 µl solution A (0.1 M Tris-HCl pH 9.0, 0.1 M EDTA and 1% SDS) and incubated at 70°C for 30 min. 28 µl 8 M KAc were added and the samples incubated for 30 min on ice. Cell debris were harvested at maximum speed for 15 min at 4°C. The supernatant was transferred to a new tube and the DNA precipitated by adding 0.5 volumes isopropanol and centrifuging at 15.000 rpm for 5 min. Pelleted DNA was washed with 1 volume 70% ethanol and centrifuged. Finally, the DNA pellet was air-dried, and re-suspended in 50 µl 1× TE by rotating o/n at 4°C. After genomic DNA extraction, the number of copies was determined by quantitative Real-Time PCR using 2 ng of DNA per reaction. Primers used for real time HeT-A_F (CCCCGCCAGAAGGACGGA) and HeT-A_R (TGTTGCAAGTGGCGCGCA) for the 3′UTR region, HeT-A Real Time Gag F (ACAGATGCCAAGGCTTCAGG) and HeT-A Real Gag Time R (GCCAGCGCATTTCATGC) for the *Gag* gene, Actin_F (GCGCCCTTACTCTTTCACCA) and Actin_R (ATGTCACGGACGATTTCACG).

### RNA extraction and cDNA synthesis

Total RNA was isolated from ten whole third instar larvae and extracted using RNeasy Mini Kit (Qiagen) according to manufacturer's protocol. RNase Free DNase Set (Qiagen) was used to remove genomic DNA contaminations as follows: one on column during the extraction accordingly to manufacturer's protocol, and two in solution for 2 hours at 37°C. RNA was cleaned by precipitation and its quality was assessed using NanoDrop spectrophotometry.

One microgram of RNA was reverse transcribed into cDNA using Transcriptor First Strand cDNA Synthesis Kit (Roche) with oligo(dT) primers, and the expression of the different transcripts analyzed by quantitative Real-Time PCR. For each fly strain, two independent RNA extractions were prepared and analyzed three independent times. Primers used for real time PCR: HeT-A_F (CCCCGCCAGAAGGACGGA) and HeT-A_R (TGTTGCAAGTGGCGCGCA) for the 3′UTR, HeT-A Real Time Gag F (ACAGATGCCAAGGCTTCAGG) and HeT-A Real Gag Time R (GCCAGCGCATTTCATGC) for the *Gag* gene. Actin_F (GCGCCCTTACTCTTTCACCA) and Actin_R (ATGTCACGGACGATTTCACG).

### Chromatin immunoprecipitation experiments (ChIPs)

Brains and imaginal discs from third instar larvae were dissected in 1× PBS with protease inhibitors. After dissection, the brains were resuspended in 5 ml buffer A (60 mM KCl, 15 mM NaCl, 15 mM HEPES pH 7.6, 0.5% Triton X-100, 0.5 mM DTT, complete EDTA-free protease inhibitor cocktail from Roche) with 1.5% formaldehyde, homogenized in a Wheaton Dounce and incubated for 15 min at room temperature. Crosslinking was stopped by adding glycine to a final concentration of 0.125 M and incubating 5 min at 4°C on a rotating wheel. Brains were washed 3 times with Buffer A and resuspended in lysis buffer (140 mM NaCl, 15 mM HEPES pH 7.6, 1 mM EDTA, 0.5 mM EGTA, 1% Triton X-100, 0.5 mM DTT, 0.1% sodium deoxycholate, complete EDTA-free protease inhibitor cocktail from Roche). Cross-linked nuclei were fragmented using bioruptor sonicator (high amplitude, 15 sec ON and 45 sec OFF). For the following steps a standard protocol (Upstate) was used. Thirty brain/discs complexes were used per IP. Chromatin was immunoprecipitated with the following antibodies: anti-H3K9me3 (ab8898, Abcam), αnti-H3K4me3 (ab8580, Abcam), αnti-HP1 (The anti-HP1 (C1A9) antibody developed by Lori L. Wallrath was obtained from the Developmental Studies Hybridoma Bank developed under the auspices of the NICHD and maintained by The University of Iowa, department of Biology, Iowa City, IA 52242), αnti-JIL-1 (gift from Kristen M. Johansen), and αnti-Z4 (gift from Harald Saumweber). Three independent ChIP samples were analyzed and the amount of immunoprecipitated DNA was calculated by quantitative real-time PCR using iQ SYBR Green Supermix (BioRad). Primers used for real time PCR: HeT-A5′UTR_F (TCGCGCTTCCTCCGGCAT) and HeT-A 5′UTR_R (GCGGTTATTACATTACGGCGG), RpL32-F (CAAGAAGTTCCTGGTGCACAA) and RpL32-R (AAACGCGGTTCTGCATGAG).

### Quantitative real-time PCR

Quantitative real-time PCR was performed to determine *HeT-A* copy number and *HeT-A* expression, and in ChIP experiments. The iQ5 Multicolor Real-Time PCR Detection System was used and the iQ SYBR Green Supermix (BioRad) was used to prepare the reactions. Relative levels of *HeT-A* expression were determined using the threshold cycle and normalized to actin levels (or RpL32 for ChIPs). Three independent experiments of two samples each strain were performed.

### Chromosome cytology and immunostaining

Third instar larvae brains were dissected in 0.7% NaCl solution, incubated in 10 µM colchicine (Roche) for 2 hours and submitted to a hypotonic shock (0.5% sodium citrate) for 10 min. Brains were fixed in 60% acetic acid and squashed. For anaphase preparation, brains were dissected as before, the hypotonic shock was omitted, and the brains were successively immersed in 45% and 60% acetic acid. DNA was stained with DAPI in mowiol medium. Mitotic chromosome preparations were analyzed on a Zeiss Imager Z2 fluorescence microscope using the AxioVision software.

### Direct visualization of GFP-fusion proteins on mitotic chromosomes

Third instar larvae brains were dissected in 1× PBS with protease inhibitors and incubated in 0.5 mg/ml colcemid (Roche) for 2 hours. A hypotonic shock was applied by incubating brains in 0.5% sodium citrate for 10 min. Proteins were fixated by incubating with Brower's Fixation Buffer (0.15 M PIPES, 3 mM MgSO4, 1.5 mM EGTA, 1.5% NP-40, and 2% formaldehyde, pH 6.9) for 3 min. Brains were washed in 1× PBS-Triton (0.1%) for 3 min and allowed to soak in 50% glycerol for 5 min. Brains were squashed in a drop of glycerol, immersed in liquid nitrogen, and mounted in DAPI-mowiol medium. Mitotic chromosome preparations were analyzed on a Zeiss Axio Imager.Z2 fluorescence microscope using the AxioVision software.

### RNAi knockdown in S2 cells

Fragments of the *Z4*, *HeT-A gag*, *hoap*, and *Sart1* (non-LTR retrotransposon from *Bombyx mori*) coding sequences were amplified by PCR and cloned in pSTBlue-1 vector to produce dsRNA. Single stranded RNAs were synthesized by using SP6 and T7 RNA polymerases (Promega), both strains were incubated for 5 min at 90°C and annealed by slowly cooling to room temperature to obtain the dsRNA. dsRNA was then precipitated and treated with DNase (Qiagen) and RNase A (Roche) for 15 min at 37°C. A phenol:chloroform extraction was performed followed by precipitation and quantification of dsRNA with NanoDrop spectrophotometer ND1000. 50 µg of dsRNA were diluted in 1 ml of supplemented Schneider medium, added drop-wise to a total of 1.5×10^7^ cells, and incubated at 25°C. The same protocol was repeated at 24 h and 48 h after seeding the cells. An aliquot of cells was collected at 24 h, 48 h, and 72 h after seeding. For description of cytology experiments, see next section S2 cells metaphase chromosome preparation. Gene fragments of about 550 bp were amplified using the primers: HeTGag-RNAi-F (CTAGCGGCAAACAACATCG) and HeTGag-RNAi-R (GGGATTGCAGATTCTTGGC) to amplify *HeT-A* sequence with accession number: X68130 from nucleotide (nt) 2701 to nt 3255, Z4-RNAi-F (TAATTATCCAGCAGGGACAG) and Z4-RNAi-R (CAATCAGATCTGGTCTTTGTCTCCGTAAAC) to amplify the *Z4* gene acc num: CG7752 from nt 3046 to nt 3383, HOAP-RNAi-F (GCCGAGACTAAGAAGCAGAAC) and HOAP-RNAi-R (CCTGATCGTCAGGCTCTTG) amplify the *caravaggio* gene acc.num: CG6219 from nt 1689 to nt 2166, and SART1-RNAi-F (CAACGGCAGCAGAATCAATG) and SART1-RNAi-R (CGTAATTTCTCCGCCAGCAA) amplify the *SART1* retrotransposon acc.number: D85594 from nt 1943 to nt 2432. All amplified regions were checked for off-site targets.

### S2 cells metaphase chromosome preparation

500 µl collected cells were treated with 500 µl colcemid (10 µg/ml; Roche) during 2–3 h in the dark. Cells were centrifuged 3 min at 1500 rpm and the pellet was washed with PBS. Cells were centrifuged again and the pellet resuspended with 500 µl 0,5% sodium citrate. After 10 min r.t. incubation, cells were centrifuged, resuspended in 1 mL fixation solution (methanol∶acetic, 3∶1) and incubated for 10 min. Cells were centrifuged again, re-suspended in 50 µl Fixation Solution, and cells were dropped onto a microscope slide. Slides were air dried and mounted in DAPI-containing Mowiol medium. Images were obtained using the Zeiss Axio Imager.Z2 fluorescence microscope.

### Cell transfection

Drosophila S2 cells were seeded at 3×10^6^ cells/ml and transfected with one microgram of plasmid DNA using Effectene Transfection Reagent (Qiagen), accordingly to manufacturers protocol. Cells were incubated for 48 hours at 25°C and collected by centrifugation at 2000 rpm for 5 min, washed twice in 1× PBS and frozen at −80°C. HeT-A Gag-GFP plasmid was used in cell transfection [37].

### Protein co-immunoprecipitation assays

Protein extracts from S2 cells were prepared in 1 ml lysis buffer (50 mM Tris-HCl pH 7.4, 100 mM NaCl, 1% TritonX-100, 1 mM EDTA, 1 mM EGTA, and Complete EDTA-free protease inhibitor cocktail from Roche), incubated on ice for 20 min, and centrifuged at 13 000 rpm for 15 min at 4°C. Fresh lysates were incubated with 50 µl PureProteome Protein A and Protein G Magnetic Beads (Millipore) coated with specific antibodies, for 4 hours at 4°C with rotation. The magnetic beads were previously incubated with the respective antibodies in 500 µl lysis buffer for one hour at 4°C with rotation and washed 3 times with 500 µl lysis buffer. Immunocomplexes were washed 3 times with lysis buffer and eluted from the beads with 50 µl 1× sample buffer. Samples were boiled for 10 min, loaded on a SDS-PAGE gel and analyzed by Western Blot. Anti-GFP (Invitrogen, A11120), anti-Z4 [58], anti-JIL-1 (mouse, gift from Kristen Johansen), and control mouse IgG (Santa Cruz Biotechnology, sc-2025) were used for protein immunoprecipitation, and anti-HeT-A Gag, anti-Z4 and anti-JIL-1 [59] were used in Western Blot experiments.

## Supporting Information

Figure S1 *HeT-A* copy number of *JIL-1* and *Z4* mutants. The genomic content of the *HeT-A* retrotransposon of each stock was measured in *HeT-A Gag* (A) and *HeT-A* 3′UTR (B) regions. *Z4^7.1^* and *Su(var)2-5^05^* mutant alleles have more *HeT-A* copies than control flies. Error bars represent standard deviations of three independent experiments. Asterisks indicate statistically significant differences using the t-test (one asterisk, *P*<0.05 to 0.01; two asterisks, *P*<0.01 to 0.001; three asterisks, *P*<0.001) in *HeT-A* copy number of each mutant compared to *w^1118^*.
(TIF)

Figure S2 *HeT-A* expression in *JIL-1* and *Z4* mutants. Absolute expression of *HeT-A gag* (A) and *HeT-A* 3′UTR (B) in the analyzed stocks. *HeT-A* transcription was normalized to actin transcription. Error bars represent standard deviations of three independent experiments.
(TIF)
