# Supplementary figures and images for: The Chromosomal Proteins JIL-1 and Z4/Putzig Regulate the Telomeric Chromatin in Drosophila melanogaster
Source: PLoS Genet. 2012 Dec 13;8(12):e1003153. doi: 10.1371/journal.pgen.1003153 (PMC3521665; doi:10.1371/journal.pgen.1003153)

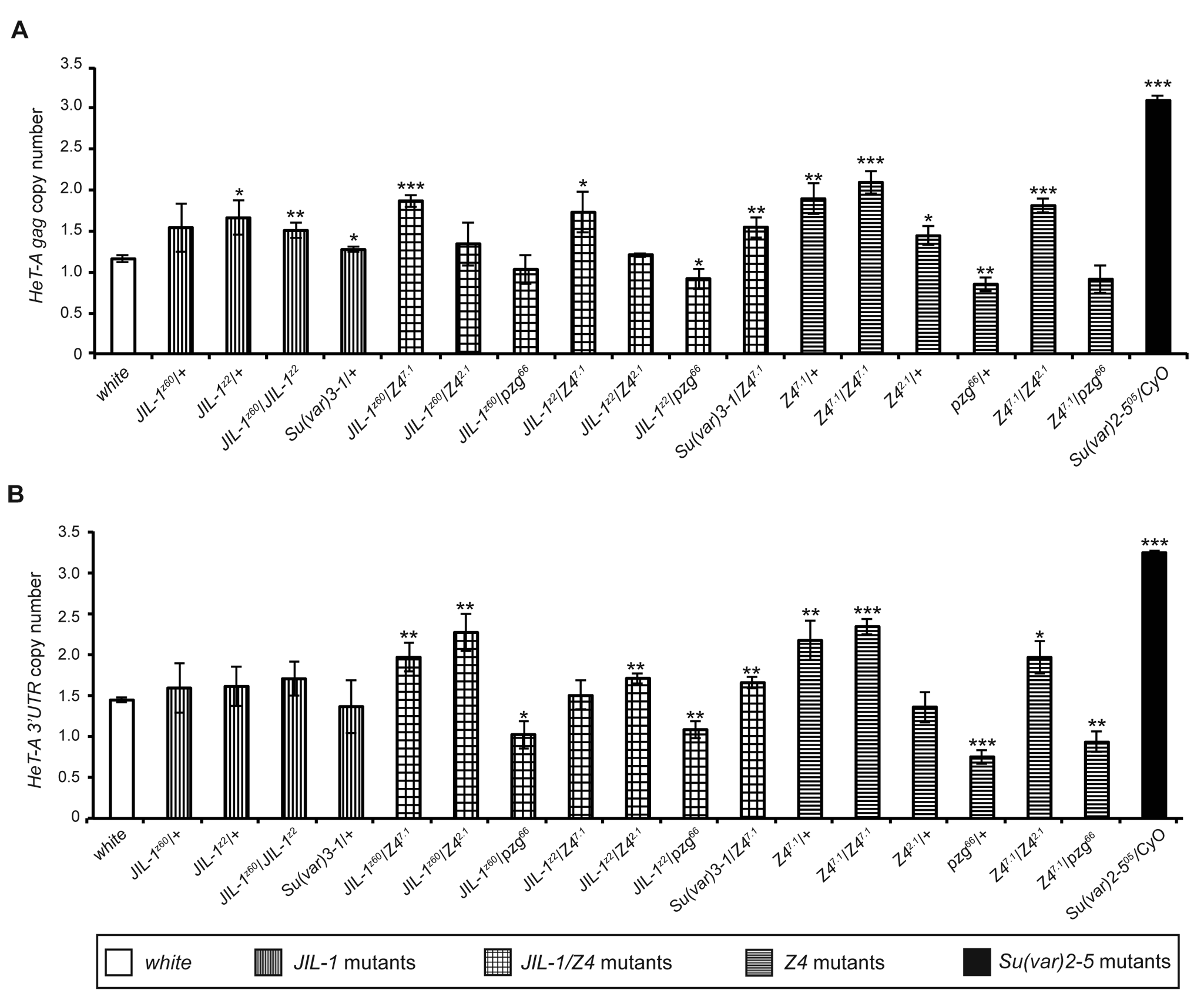

Supplement: Figure S1 — HeT-A copy number of JIL-1 and Z4 mutants. The genomic content of the HeT-A retrotransposon of each stock was measured in HeT-A Gag (A) and HeT-A 3′UTR (B) regions. Z47.1 and Su(var)2-505 mutant alleles have more HeT-A copies than control flies. Error bars represent standard deviations of three independent experiments. Asterisks indicate statistically significant differences using the t-test (one asterisk, P<0.05 to 0.01; two asterisks, P<0.01 to 0.001; three asterisks, P<0.001) in HeT-A copy number of each mutant compared to w1118. (TIF) [file pgen.1003153.s001.tif]

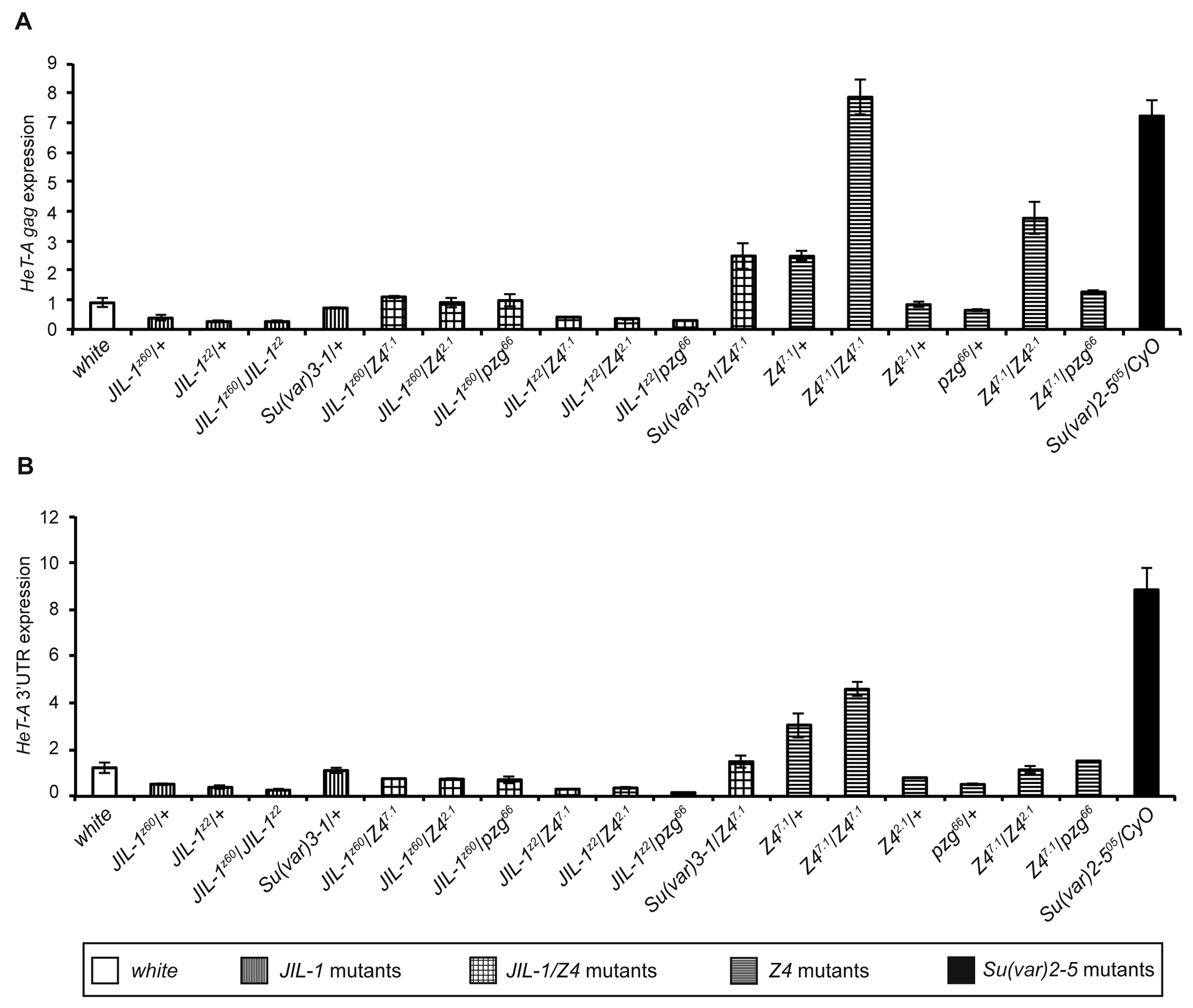

Supplement: Figure S2 — HeT-A expression in JIL-1 and Z4 mutants. Absolute expression of HeT-A gag (A) and HeT-A 3′UTR (B) in the analyzed stocks. HeT-A transcription was normalized to actin transcription. Error bars represent standard deviations of three independent experiments. (TIF) [file pgen.1003153.s002.tif]
